# Exploring the role of goal setting in weight loss for adults recently diagnosed with pre-diabetes

**DOI:** 10.1186/s12912-020-00462-6

**Published:** 2020-07-15

**Authors:** Lisa Whitehead, Courtney C. Glass, Sally L. Abel, Kiri Sharp, Kirsten J. Coppell

**Affiliations:** 1 School of Nursing and Midwifery, Edith Cowan University, 270 Joondalup Drive, Joondalup, 6027 Australia; 2 Kaupapa Consulting Ltd, Napier, 4110 New Zealand; 3 Edgar Diabetes and Obesity Research, Department of Medicine, Dunedin School of Medicine, University of Otago, PO Box 56, Dunedin, 9054 New Zealand

**Keywords:** Diet therapy, Goals, Indigenous population, Prediabetes, Practice nurses, Type 2 diabetes, Weight loss

## Abstract

**Background:**

The management of prediabetes in the community setting is a global priority. We evaluated the feasibility of a 6-month multilevel practice nurse-led prediabetes dietary intervention which involved goal setting. The aim of this paper is to explore the weight loss goals and strategies reported by participants to achieve their weight loss goals as recorded by practice nurses, and report on factors that influenced dietary behaviours.

**Methods:**

This study used a convergent mixed-methods design. A six-month pragmatic non-randomised pilot study with a qualitative process evaluation was conducted in two neighbouring provincial cities in New Zealand. A structured dietary intervention delivered by practice nurses was implemented in four practices in 2014–2016. Content analysis of the text and descriptive statistics were used to analyse the data.

**Results:**

One hundred and fifty seven people with prediabetes were enrolled (85 intervention, 72 control). The intervention group lost a mean 1.3 kg more than the control group (*p* < .0.001). The majority of the intervention group indicated either a high level of readiness (*n* = 42, 53%) or some readiness (*n* = 31, 39%) to make food changes. The majority of weight loss goals aligned with clinical guidelines (between 5 and 10% of body weight). While just over half (*n* = 47, 55%) demonstrated weight loss at the end of the six month period, the majority of participants did not achieve their predetermined weight loss goal (*n* = 78, 83%). Gender, ethnicity and budget were not related to weight loss at six months. Readiness to change and reported challenges to making dietary changes were related to weight loss at six months. Negative factors or set-backs included sporadic adherence to diet due to other health problems, change in context or environment and coping with ill health, most notably stress and low mood.

**Conclusions:**

The data relating to weight loss and dietary goals provided insight into the challenges that people faced in making dietary changes for weight loss across a six month period. Simplifying goal setting to those goals with the greatest potential clinical impact or the greatest significance to the person, in a socially supportive environment, may increase the success of goal achievement.

**Trial registration:**

ANZCTR ACTRN1261500080656. Registered 3 August 2015 (Retrospectively registered). https://www.anzctr.org.au/Trial/Registration/TrialReview.aspx?id=366560&isReview=true

## Background

Prediabetes is a condition that indicates a sustained level of hyperglycaemia that is lower than the threshold for type 2 diabetes mellitus (T2DM). Globally, prevalence estimates of prediabetes vary according to the test and diagnostic criteria used [1]. In New Zealand and the US where glycated haemoglobin (HbA1c) is used, the prevalence of prediabetes among the adult population is around 25% [1–3]. Of those with prediabetes, each year an estimated 5–10% develop T2DM [4, 5] with the majority eventually developing the condition, particularly those who are overweight or obese [6].

The diagnosis of prediabetes presents an opportunity to make lifestyle changes, including weight loss, to achieve regression to normoglycaemia [7]. Nutrition is important in the management of many chronic conditions and a key mediator of weight management and glycaemic control in prediabetes and the prevention of T2DM [8]. The incorporation of information and support around nutrition and diet has been described as severely lacking in clinical practice [9], and a missed opportunity for nurses [10]. In addition to the issues of the provision of evidence-based nutritional advice are the multifaceted issues around behaviour change and the ability to uptake lifestyle advice.

Goal setting has been promoted as motivating behaviours that direct attention and action, mobilize short and long term effort and motivate the individual to choose relevant strategies to accomplish their goals [11]. It has been described as an important motivational factor underpinning change in health-related behaviour [12]. The mechanisms by which goal setting at diagnosis are related to future weight and glycaemic control among people diagnosed with prediabetes is unclear where mediating factors between goal setting and outcome remain largely unexplored.

The Prediabetes Intervention Package (PIP) study explored the impact and cost-effectiveness of a dietary intervention in which practice nurses working in primary health care centres in community settings facilitated goal setting and provided dietary advice and support for people recently diagnosed with prediabetes on the outcomes of weight and glycaemic control [7, 13]. This paper focusses on influences, processes and outcomes in relation to goal setting as recorded by the practice nurses during consultations with participants.

## Methods

### Setting and study design

A mixed-methods design involving a non-randomised intervention pilot with quantitative and qualitative evaluation was developed a priori the commencement of the study, to assess the effectiveness of the intervention. A detailed description regarding the methodology, study design and further results have been published [7]. Participants were followed-up over a 6 month period to assess weight and HbA1c. Semi-structured interviews were conducted with a purposive sample of 20 participants, and 17 key informants including practice nurses. The study was conducted in general practices and community settings in two neighbouring provincial cities in Hawke’s Bay, New Zealand. Indigenous Māori have high rates of prediabetes and T2DM, and between 18.2 and 23.0% of these cities were Māori [2, 14]. Participant characteristics were similar between control and intervention and within the intervention group. Inclusion criteria were non-pregnant adults aged 70 years or less, newly diagnosed with prediabetes (within 6 months of diagnosis; HbA1c 41–49 mmol/mol or fasting plasma glucose 6.1–6.9 mmol/L [15]), classified as overweight or obese (≥25 kg/m^2^), able to communicate in English and not prescribed Metformin, an oral medication commonly used to lower blood sugar levels.

A practice nurse completed a follow-up phone call and appointments were made with those willing to participate to discuss the study further. Participant recruitment occurred between August 2014 and April 2015 and data collected 2014–2016. The aim of this paper is to explore the weight loss goals and strategies reported by participants to achieve their weight loss goals and recorded by the practice nurses at baseline and the follow-up visits.

### Intervention

The aims of the intervention were to develop knowledge of healthy eating and to support and empower participants to make dietary changes [7]. Training was provided for the practice nurses consisting of a six-hour evidence-based course that included nutrition principles, goal setting, dietary assessment, how to provide individualised nutritional advice and the completion of anthropometric measurements. This was delivered by the study investigators and a local dietitian. The dietitian and liaison nurse arranged review sessions on a monthly basis to support intervention adherence and provide support.

The intervention began with a 30-min session with a practice nurse on dietary behaviours. Participants were asked to complete a brief dietary assessment form before the session. Starting the Conversation:Diet (STC) was utilised in this study, with minor alternations to account for the New Zealand setting [7, 16]. This tool is a validated eight-item simplified food frequency questionnaire specifically designed for primary care and health promotion settings. The psychometric testing reported that the eight STC items and summary score performed well. STC items and the summary score were moderately intercorrelated (*r* = 0.39–0.59, *p* < 0.05) [16]. The STC summary score was significantly correlated with the NCI fat screener at baseline (*r* = 0.39, *p* < 0.05), and change in the STC summary score correlated with reduction in percentage of calories from fat (*r* = 0.22, *p* < 0.05) from baseline to 4 months [16]. The STC was sensitive to the intervention, with intervention participants improving significantly more than controls on the summary score (*M* = 1.16 vs 0.46, *p* < 0.05) [16].

The STC was used in conjunction with sociocultural contextual questions (e.g. household budget, purchased foods, specific dietary requirements) and anthropometric measurements (weight, height and waist circumference). A Detailed Dietary Assessment (DDA) was completed to explore unhealthy dietary habits identified in the STC:Diet and the data used to facilitate the goal setting process. At the first session, participants set three nutritional goals and a weight loss goal of 5–10% of body weight over 6 months with the practice nurse. The decision to use a 5–10% weight loss goal was based on the lifestyle intervention goals of the Diabetes Prevention Program which demonstrated lifestyle advice prevents progression from prediabetes to T2DM [17] and in recognition of the strong scientific evidence that gradual weight loss of 5–10% over a 6 month period is more sustainable long term and that a 5–10% weight loss is linked to many health benefits [17]. The goals were recorded in the patient management system to allow general practitioners to view the information and reinforce goals at appointments. Participants were asked at baseline to self-report readiness to make food changes on a 1–10 point scale (with 1 being not ready at all and 10 being really motivated). This scale was developed for the study and is available as a supplemental file. Follow-up appointments were arranged at 3 weeks, 3 months and 6 months after the initial appointment and goals were reviewed and updated accordingly. Implementation fidelity was high [7]. All nurses attended training and update sessions and were assessed as delivering the brief dietary assessment, goal setting and appropriate dietary advice as per the study protocol.

One hundred and 57 patients with prediabetes were enrolled (85 intervention, 72 control) and weight loss differences between the intervention and control group were significant, with an adjusted mean weight loss of 1.30 kg (*p* < 0.001) at 6 months.

This paper presents the findings recorded by the practice nurses in relation to weight loss goals, strategies to achieve weight loss goals and feedback on any factors participants described as influential on their ability to meet their goals.

### Data analysis

The outcomes of interest for this study were the impact of goal setting on weight. Socio-demographic factors were explored in relation to weight and content analysis of the notes recorded by the practice nurses completed to assess weight loss goals and strategies recorded to achieve these. The Statistical Package for Social Scientists (SPSS version 24) was utilised for quantitative analysis of data. Paired sample t-tests were utilised to assess the mean change over time within each outcome variable. Chi-square tests, fisher’s exact tests and one-way ANOVA tests were completed to determine any significant differences in primary and secondary outcomes between different socio-demographic populations, within the intervention group. Qualitative assessment of goal setting was completed through content analysis in Microsoft Excel 2016. Specifically, data related to the nutritional goals and participant feedback on factors that had influenced their ability to achieve goals were analysed through content analysis [18]. Findings were assessed for similarities and grouped into categories. The analysis was reviewed and discussed by the whole research team to enhance rigor of analysis and reporting.

## Results

The mean age of the participants (*n* = 85) in the intervention group was 59 years (± 9.1 years). The majority of participants were New Zealand European/Pākehā (*n* = 52, 61%) and just under a third (*n* = 27, 32%) identified as indigenous Māori. A high proportion (*n* = 34, 40%) had a family history of T2DM. The majority of participants were the household’s grocery shopper (67%) and the cook in the household (68%). Almost half (47%) the sample reported they had a limited budget for food and nearly one third (*n* = 26, 31%) lived alone. Self-reported readiness to make food changes ranged from 2 to 10 (on a 0–10 point scale). Just over half the sample indicated a high level of readiness to make food changes (*n* = 42/79, 53%), 31 indicated some readiness to make food changes (39%) and a small number indicated low readiness to make food changes (*n* = 6, 8%). Data were missing for six participants. At baseline, participants identified as obese were more likely to report a limited budget for food compared to overweight participants (*p* = 0.024).

Of the 66 weight loss goals recorded at baseline, 59 were classified as aligning with the clinical guidelines (between 5 and 10% of body weight) and six were classified as not. Of the six, five goals were just over 10% of total body weight and one was 20% of body weight. The majority of weight loss goals remained the same across the 6 month period (*n* = 46) with seven participants choosing to increase their weight loss goal over time and 13 participants decreasing their weight loss goal.

Budget, age, gender and ethnicity were not related to setting a weight loss goal of 5–10% nor to setting a weight loss goal at each follow-up point (1 month, 3 months and 6 months). The majority of participants did not achieve their weight loss goal (*n* = 78, 82.7%). There were no significant differences between setting a weight loss goal of 5–10% and achieving the weight loss goal.

Although only seven participants met their weight loss goal, just over half of participants (*n* = 47, 55%) demonstrated weight loss at the end of the 6 month period. Gender, ethnicity and budget were not related to weight at 6 months. The pattern of weight loss was either consistent (*n* = 31) or variable, going both up and down over the course of the 6 month period, with a final weight reduction at 6 months (*n* = 16). The remainder either maintained a consistent weight over the 6 month period (*n* = 12) or an increase in weight (*n* = 26), classified as a consistent increase in weight (*n* = 8) or variable over the course of the6month period (*n* = 18).

A pattern in readiness to change was associated with weight loss at 6 months. Of those who reported a consistent decrease in weight, over half (59%, *n* = 16) indicated a high readiness for change versus 13% (*n* = 1) of those who experienced a consistent increase in weight over the 6 month period.

In addition to a weight loss goal, participants determined nutritional goals with their practice nurse. The number of goals ranged from 0 to 7 and setting three goals was most common and three participants did not set a nutritional goal. The goals were categorised into twelve categories (Table 1).

**Table 1:** Nutritional goals by category

Category	N
Increase vegetable/fruit intake	50
Reduce carbohydrate intake (amount and types of carbohydrate consumed).	43
Cut back on high fat foods (e.g. cheese, butter, choosing leaner meats)	36
Monitor portion size	35
Choose healthier food options (healthier snack options)	28
Reduce amount of sweetened beverages (including added sugar to tea and coffee)	28
Decrease the consumption of sweets	23
Increase water consumption	22
Switch to healthier takeaway options/limit takeaways	16
Reduce alcohol consumption	13
Eat regularly	11
Increase fish/chicken consumption	11

The most common nutritional goals were to increase fruit and vegetable consumption, followed by reducing carbohydrate consumption, cutting back on high fat foods and monitoring portion size.

The first three goals as set at baseline were assessed against the criteria of the acronym SMART (Specific, Measurable, Attainable, Realistic, Timeline). Goals that contained all elements were assessed as a “SMART” goal and those that did not were recorded as “not SMART”. An example of a SMART goal was “Add two servings of vegetables to the evening meal per day from the non-starchy list”. An example of a goal that did not meet the SMART criteria was “Portion sizes to decrease”. Twenty eight participants (33%) set three SMART goals at baseline and 20 (24%) participants set two SMART goals (and one “not SMART” goal). Twenty (24%) participants had set one goal that was assessed as meeting the criteria of SMART and eight (9%) participants had not set any SMART goals. No relationship between setting SMART goals and weight change at 6 months was noted.

The practice nurses followed up participants at 3 months and 6 months and recorded feedback on how the participant felt about the goals and anything that participants described as influencing their weight or glycaemic control. Sixteen (19%) participants reported positive changes and provided feedback on working to achieve their goals. Fifteen (18%) participants reported a mixture of positive and negative factors and 19 (22%) reported only negative factors. Information was not recorded for 29 participants. Positive factors that supported working towards or achieving goals were classified as achieving goals e.g. eating breakfast every morning. Negative factors or set backs were classified as sporadic adherence to diet due to health problems (reported by 10 people (12%)), change in context or environment (*n* = 8, 9%) (e.g. visiting family or family coming to stay, job under threat or job loss) and coping with stress and ill health (*n* = 14, 16%), most notably stress and low mood. A pattern was noted between the report of positive, mixed and negative factors and a consistent decrease or increase in weight. Although the numbers are small, only one fifth of those who lost weight consistently over the 6 month follow-up reported experiencing negative factors compared to four fifths of those who increased in weight consistently over the6 month period.

## Discussion

In this study the majority of participants were able to set weight loss goals, construct a plan and monitor their progress however, a significant association was not found between these factors and weight loss outcome. A pattern did emerge between weight loss outcome and the report of experiences described as impacting on the ability to maintain behaviours at the 6 month follow-up. This finding is consistent with earlier work that the report of perceived obstacles and distractions are related to weight loss at follow-up [19, 20]. Whilst setbacks and challenges are highly likely to be experienced, the evidence suggests that the ability to plan and action alternative solutions and change patterns of behaviour are also important [21]. Building in an approach to problem solving and generating alterative solutions could help to support people to achieve weight loss goals.

Behaviour change is recognised as a process and one that requires different approaches to goal setting, action-planning and level of support depending on where in the process an individual finds themselves [22]. Interventions are frequently designed with the notion that participants are ready to change, however where this is not the case, a different approach may be required to facilitate engagement before goals are set. Further understanding of how goal setting and action planning can be individualised to participants’ stage of readiness to change and motivations to engage in goal setting are encouraged.

Psychological orientation to the drivers behind goals has been reported as influential in weight loss, and a tension has been reported between wanting to lose weight and the perception of having to lose weight [23]. In this study, the association between weight and prediabetes was the reason for inviting people onto the study and starting the conversation about weight loss. Whilst drivers for engaging in the study were not specifically discussed between participants and the practice nurses, independent interviews with participants confirmed that reversing prediabetes and preventing the onset of T2DM were the key reasons for engaging in the intervention, indicating the desire to improve health [20]. What was not clear in the data was whether participants saw the need to lose weight as a “want to” goal rather than a “have to” goal. In self-determination theory, the concept of autonomy (or self-determination) is central to understanding goal pursuit and why not all goals are created equal [24]. Feeling autonomous, effective, optimally challenged, and meaningfully connected to others have intrinsic value to the self and are essential for wellbeing and behavioural persistence [25]. It was evident in the data that nurses were working collaboratively with participants to develop and adapt goals that reflected participants’ circumstances and resources in relation to time, budget, access and household. These are important findings and directly relate to the importance of being optimally challenged and improving self-efficacy [26, 27]. Practice nurses sought to increase participants’ sense of self-efficacy by approaching the creation of goals to the resources that participants had the potential to control or resources they had access to, for example budget and access to affordable food, especially fruit and vegetables.

An area not specifically considered in this study but one that is gaining momentum, is the value of Peer Support to sustain health behaviors in prevention or disease management. Peer Support is defined as emotional, motivational and practical assistance provided by nonprofessionals for complex health behaviors. However, we have identified that supportive relationships (both professional and personal) were key factors in empowering participants and facilitating their ability to make dietary changes [20]. A recent review [28] with a focus on diabetes prevention and management reviewed 65 studies and reported that the majority (*N* = 54, 83.1%) reported a statistically significant and positive impact of Peer Support on health behaviours. Of the 30 studies reporting on HbA1c, the average reduction was 0.76 mmol/l. Peer support is an area that merits further exploration in the field of diabetes prevention and management, and which we are currently exploring in a follow-up study [29].

### Implications for practice

The study demonstrated that practice nurse support to set weight loss and dietary goals was both achievable and resulted in weight loss for the majority of the participants. Ensuring that the number of goals set are kept to a manageable number, may enhance goal adherence. A focus on up to three goals that are strongly aligned with client preference, may led to greater goal achievement. Ensuring that any weight or clinical outcomes that have resulted from dietary change are framed within overall well-being may be a powerful approach for nurses. Time at subsequent appointments can be focussed on reviewing current goal achieving behaviours, providing encouragement, and discussing how to overcome or minimise barriers. Discussing how to revise or modify current goal behaviours may also be beneficial for those people struggling with goal achievement.

Future research should also include in-depth interviews or focus groups to elicit “stories” about the goal setting process; tracking goal progress prospectively with a series of checks such as “same goal,” “revised goal,” or “made new goal;” and closer examination of goal selection, readiness to change and motivation to change. Simplifying goal setting to those goals with the greatest potential clinical impact or the greatest significance to the person, in a socially supportive environment may increase the success of goal achievement for people who are overweight or obese and recently diagnosed with prediabetes.

## Conclusion

The study supports the notion that clinicians, in this case practice nurses based in the primary health care setting, can engage in dietary counselling and effect positive change. Sociodemographic factors related to ethnicity, gender, age and income were not found to be directly associated with achieving weight loss. The experience of setbacks that impacted on the ability to follow goals were related to weight loss achievement. Regular follow-up through face to face and telephone appointments were highlighted as valuable and supportive. Areas for consideration are the assessment of readiness to make change, the drivers behind weight loss goals and the ability of individuals to problem solve to manage perceived setbacks. Nutrition and weight are important core components of the management of many chronic diseases and the potential gains of providing support in the primary health care setting are substantial.

## Supplementary information

**Additional File 1.** Readiness to make food changes. This file contains the measure of readiness to make food changes.

## Data Availability

Data can be made available from the corresponding author on reasonable request.

## Abbreviations

DDA: Detailed dietary assessment
HbA1c: Glycated haemoglobin
PIP: Prediabetes intervention programme
STC: Starting the conversation
T2DM: Type 2 diabetes mellitus
