# Supplementary material for: Exploring the role of goal setting in weight loss for adults recently diagnosed with pre-diabetes
Source: BMC Nurs. 2020 Jul 15;19:67. doi: 10.1186/s12912-020-00462-6 (PMC7362527; doi:10.1186/s12912-020-00462-6)
Supplement: Supplementary file 1 — Additional File 1. Readiness to make food changes. This file contains the measure of readiness to make food changes. [file 12912_2020_462_MOESM1_ESM.docx]

**Supplemental File**

**Readiness to make food changes**

On a scale of 1 – 10, how ready are you to make food changes (1 – not ready at all; 10 – really motivated)? ________________
